# Risky decision-making in the balloon analogue risk task – the role of noradrenaline and cortisol

**DOI:** 10.1007/s00213-025-06943-3

**Published:** 2025-11-10

**Authors:** Kim Fricke, Nina Alexander, Thomas Jacobsen, Henriette Krug, Kai Wehkamp, Susanne Vogel

**Affiliations:** 1 Department of Psychology, Medical School Hamburg, Am Kaiserkai 1, 20457 Hamburg, Germany; 2 Medical School Hamburg, ICAN Institute for Cognitive and Affective Neuroscience, Am Kaiserkai 1, 20457 Hamburg, Germany; 3 Department of Psychiatry and Psychotherapy, Philipps University Marburg, Rudolf-Bultmann-Str. 8, 35039 Marburg, Germany; 4 Center for Mind, Brain and Behaviour, Philipps University Marburg, Hans-Meerwein-Str. 6, 35032 Marburg, Germany; 5 Experimental Psychology Unit, Helmut-Schmidt-University/University of the Federal Armed Forces Hamburg, Holstenhofweg 85, 22043 Hamburg, Germany; 6 Faculty of Health Sciences, Medical School Hamburg, Am Kaiserkai 1, 20457 Hamburg, Germany

**Keywords:** Human, Yohimbine, Hydrocortisone, Noradrenaline, Cortisol, Testosterone, Estrogen, Risk-taking

## Abstract

**Rationale:**

Stress can significantly alter decision-making, often promoting riskier choices. While prior research suggests a link between acute stress and increased risk-taking, the specific contributions of cortisol and noradrenaline—two key neuroendocrine stress mediators—remain unclear.

**Objectives:**

Investigate how the pharmacological manipulation of two key neuroendocrine stress mediators (cortisol and noradrenaline) influences risk-taking behavior in healthy individuals.

**Methods:**

In a double-blind, randomized study, we examined the individual and combined effects of hydrocortisone (20 mg) and yohimbine (20 mg; an α₂-adrenergic receptor antagonist) on risky decision-making in 96 healthy adults (48 women, 48 men). Participants completed the Balloon Analogue Risk Task (BART) before and after drug administration, allowing for within-subject comparisons relative to individual baselines.

**Results:**

While the pharmacological manipulation effectively elevated cortisol concentration and salivary alpha amylase activity (as proxy for noradrenaline levels), no substantial effects on risk-taking behavior emerged, contrasting with some earlier findings. Exploratory analyses of associations with personality traits (anxiety, aggression, sensation seeking) and baseline levels of testosterone and estradiol revealed only weak and inconsistent relationships.

**Conclusions:**

These findings underscore the complexity of stress effects on decision-making and suggest that pharmacological activation of stress systems may not straightforwardly mimic psychosocial stress. Methodological factors such as timing, dosage, receptor specificity, and task familiarity likely play critical roles in modulating these effects.

**Supplementary Information:**

The online version contains supplementary material available at 10.1007/s00213-025-06943-3.

## Introduction

Evaluating potential benefits and losses plays an important role when making decisions. As many decisions are made under stress, for example, in high-pressure work environments, (medical) emergencies, or during a personal crisis, understanding how stress affects decision-making is important to prevent problematic consequences that may arise in the process. For instance, growing evidence suggests that stress impairs decision-making by increasing reward sensitivity and promoting riskier choices (Starcke and Brand 2016). One potential explanation could be that under stress, individuals may rely more heavily on automatic responses rather than deliberate, rational processes (Arnsten 2009; Vogel et al. 2016), leading to more impulsive decisions. In turn, impulsive, i.e., generally more risky decision-making, can lead to engagement in activities that are potentially harmful or dangerous. Since stress is thought to contribute to both the development and maintenance of disorders, in which maladaptive risk-taking is relevant, such as substance use disorders (Koob et al. 2014) or borderline personality disorder (Ghinea et al. 2019), understanding how stress and risk-taking interact is relevant for elucidating mechanisms underlying stress-related mental disorders. While the link between acute stress and risky decision-making seems to be sufficiently established (albeit with small effect size; Starcke and Brand 2016), the underlying mechanisms of how the stress response and major stress mediators affect risky decision-making are not yet sufficiently understood.

Stress has been shown to be a complex, multi-pathway phenomenon with intricate dynamics involving many biological mediators and associated psychological processes (Joels and Baram 2009). Two major pathways of the stress response that are often investigated in the context of cognitive functioning are the sympathetic nervous system (SNS) and the hypothalamic–pituitary–adrenal (HPA) axis (Ulrich-Lai and Herman 2009). Alterations in both noradrenaline (the primary effector neurotransmitter of the SNS) and cortisol (the primary effector hormone of the HPA axis), have been associated with changes in risky decision-making (e.g., Buckert et al. 2014; Van Den Bos et al. 2014), although findings were heterogeneous. For instance, elevated cortisol levels through pharmacological hydrocortisone administration have resulted in inconclusive effects, e.g., increasing risk-taking in men, but not women (Kluen et al. 2017) or, conversely, decreasing risk-taking in men without affecting loss aversion (Metz et al. 2020). In contrast, noradrenergic stimulation through administration of the α₂-adrenergic receptor antagonist yohimbine had no effect on risk-taking (Metz et al. 2020). Regarding studies using yohimbine, it is important to note that while yohimbine stimulates noradrenaline transmission on presynaptic α₂-adrenergic receptors, it concurrently reduces noradrenaline transmission through postsynaptic mechanisms (albeit to a much lesser degree; Borowski et al. 1977; Starke et al. 1975). Thus, the net effect on noradrenaline seems to be an increase in neurotransmission. Additionally, it should be kept in mind that α₂-adrenergic receptors in turn can act as presynaptic heteroceptors on serotonergic and dopaminergic neurons (Grenhoff et al. 1995; Numazawa et al. 1995), thereby also altering serotonin and dopamine concentrations. Thus, while we will refer to yohimbine as a drug increasing noradrenergic drive in this manuscript, its pharmacology is more complex and may involve additional effects. Another study in male participants suggested that the combined administration of hydrocortisone and yohimbine (but not either drug alone) may reduce loss aversion, but not risk attitude, resulting in an alignment of reward-sensitivity with loss-sensitivity (Margittai et al. 2018). This points to a potential interaction between noradrenaline and cortisol, an effect that has also been observed in other cognitive domains including memory consolidation (Barsegyan et al. 2010, 2019; Quirarte et al. 1997; Roozendaal et al. 2006b), instrumental learning (Schwabe et al. 2010; 2012), and fear conditioning (Roozendaal et al. 2006a). The limited available research shows that the underlying mechanism of how the stress response and major stress mediators affect risky decision-making are not yet sufficiently understood and require further investigation (for a systematic review, see Sarmiento et al. 2024).

One task that has been utilized in many studies on risk-taking is the Balloon Analogue Risk Task (BART) in which participants are required to blow up a virtual balloon by mouse click. Each click increases the potential monetary reward, but if the balloon bursts, they lose all accumulated earnings (Lejuez et al. 2002). The BART has been shown to be sensitive to acute stress, sometimes resulting in gender-specific effects with increased risk-taking in men and reduced risk-taking in women (Lighthall et al. 2009; for contrasting evidence see Molins et al. 2022). Whereas pharmacological stimulation of the mineralocorticoid receptor, one of two cortisol receptor types, led to increased risk-taking independent of sex (Deuter et al. 2017), Kluen et al. (2017) reported increased risk-taking only in men following hydrocortisone administration, which activates both mineralocorticoid and glucocorticoid receptors. Yohimbine had no effect in that study, underscoring the need for further research.

To investigate how the stress mediators cortisol and noradrenaline affect risky decision-making, we used a double-blind, randomized, but gender-balanced design with pharmacological modulation (either hydrocortisone, yohimbine, both, or placebo). The BART was performed before and after drug administration, establishing a baseline before investigating the effects of hydrocortisone and yohimbine on risky decision-making and thereby increasing statistical power to detect medication effects. We hypothesized that combined hydrocortisone and yohimbine would increase risk-taking, similar to acute stress as seen in the literature (Starcke and Brand 2016). We further examined whether these effects were moderated by gender or baseline levels of testosterone and estradiol (as suggested, e.g., by Kurath and Mata 2018), and explored associations with specific personality traits, i.e., trait anxiety, trait aggression, and sensation seeking, as similar traits were associated with BART outcome measures in the past (Reynolds et al. 2013; e.g., Wise et al. 2015; Zhu et al. 2023).

## Material and methods

### Participants and experimental procedures

The sample investigated in this study was previously described by Fricke et al. (2023), who offer a detailed overview of recruitment and experimental procedures. In brief, the experiment was completed by ninety-six healthy individuals (48 self-identified men, 48 self-identified women, age: 18–35 years, M = 24.69, SD = 4.47) with no history of conditions known to affect HPA axis or sympathetic nervous system functioning, for example, endocrine or mental disorders. They were instructed to arrive well-rested, limit themselves to light physical exercise on the day of their laboratory visit, avoid the use of psychoactive substances starting the day prior to the experiment, have a light meal roughly 2 h before the experiment, and avoid food and drink intake (besides water) in the 30 min leading up to the experiment. To avoid effects of the menstrual cycle on stress reactivity, women additionally needed to have a regular menstrual cycle (e.g., excluding pregnancy, breast-feeding, or use of hormonal contraceptives) and were scheduled for the laboratory visit during their luteal phase (Kirschbaum et al. 1999). The target sample size of 96 was determined via an a-priori power analysis, assuming an alpha level of 0.05, a power of 80%, and the detection of medium-sized effects in repeated-measures ANOVAs with four between-subject groups (G*Power 3.1.9.7; Faul et al. 2007). Due to non-compliance during the experiment, two male participants were excluded from analyses, resulting in a sample size of 94. Participants provided written informed consent and received monetary compensation (30 Euro; or 5 Euro and partial course credit) for participation. The study was approved by the local ethics committee (Ethik-Kommission der Ärztekammer Hamburg; PV 5310).

To determine how hydrocortisone, yohimbine, and their interaction affect risk-taking behavior, participants were pseudo-randomly assigned to four gender-balanced groups in a fully crossed double-blind design, and received 1) 20 mg hydrocortisone and placebo (*n* = 23), 2) 20 mg yohimbine and placebo (*n* = 23), 3) 20 mg hydrocortisone and 20 mg yohimbine (*n* = 24), or 4) placebo (*n* = 24).

**Fig. 1:**
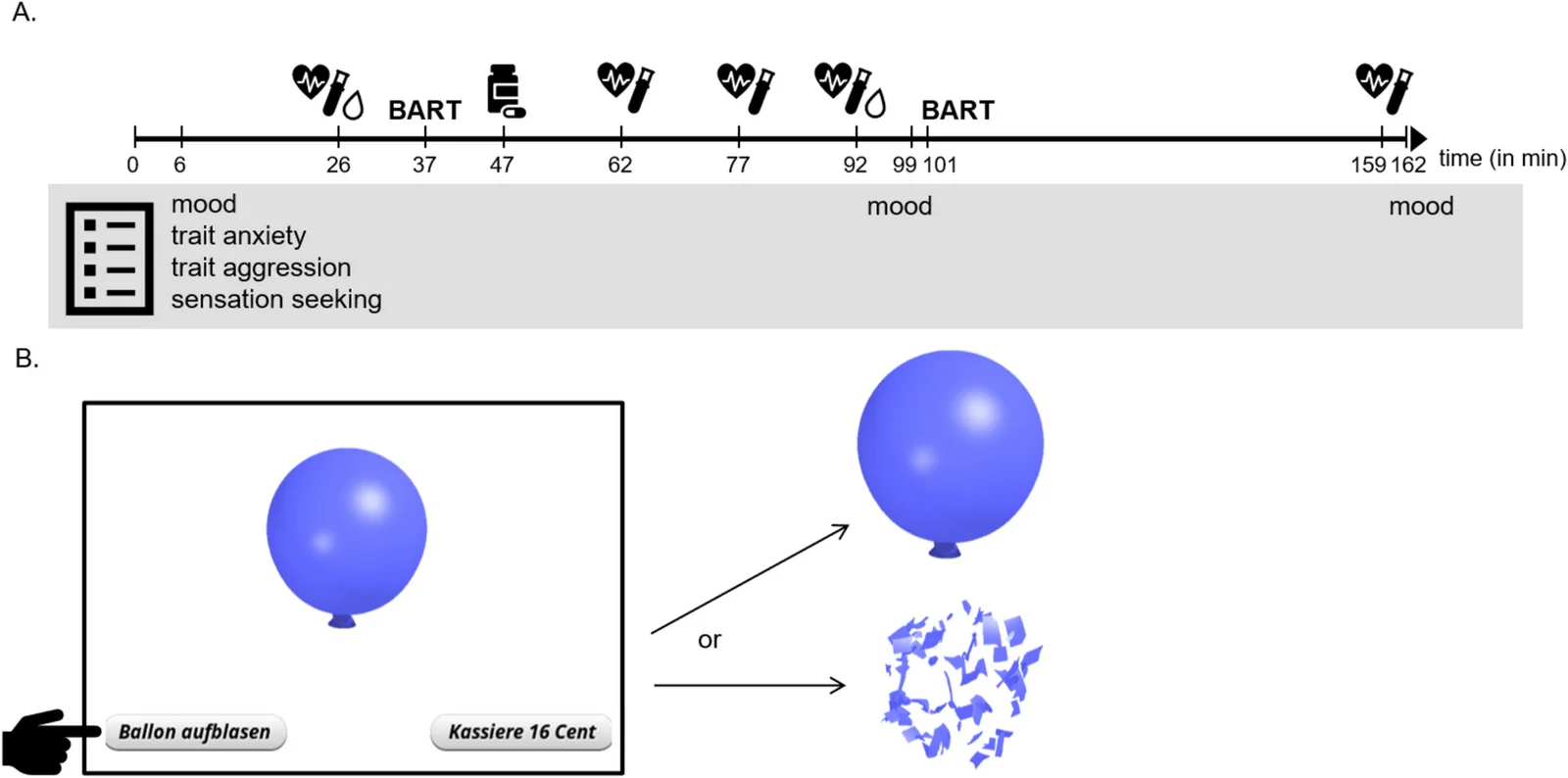
**A** Experimental design of the study. The indicated real timings (in minutes) are averaged over all participants. Relevant self-reported constructs are displayed in the gray box, while balloon analogue risk task (BART) runs, physiological measurements, and pharmacological intervention are indicated above the timeline. The medication dispenser represents the pharmacological manipulation; heart and salivette icons represent vital signs measurements and saliva sampling for cortisol and alpha amylase assessment; the droplet represents additional native saliva sampling. **B** Example screen of the BART (left) and potential outcomes (right). The left button allows the participant to blow the balloon up, while the right button allows cashing out, ending the trial. The left button can also cause bursting of the balloon based on predefined likelihoods. Translations: Left button: Blow up balloon; right button: Collect 16 cents

The experimental procedure is illustrated in Fig. 1A. After arriving at the laboratory, participants answered questionnaires assessing current mood (MDBF; Steyer et al. 1994, and three items regarding subjective stress levels (feeling anxious, upset, stressed, each answered on a scale from 1 to 9)), trait anxiety (STAI-T; Laux et al. 1981), chronic stress (TICS; Schulz et al. 2004; not reported here), trait aggression (DAF; Werner and von Collani 2014), and sensation seeking (SSS-V; Beauducel et al. 2003). This was followed by a baseline saliva sample (T1) to assess both cortisol concentrations as index of the HPA axis and alpha amylase activity as marker of noradrenergic activity using Salivettes® (code blue, Sarstedt, Germany). At the same time, vital signs (heart rate, diastolic, and systolic blood pressure) were assessed with an automated wrist monitor (RS2, OMRON, the Netherlands). In addition, native saliva was collected via microtubes with a straw to assess endogenous testosterone and estradiol concentrations.

After baseline measurements, participants completed the first BART (Lejuez et al. 2002, see below), and then received the assigned pharmacological intervention orally. Afterwards, cortisol, salivary alpha amylase, and vital signs were assessed every 15 min for a total of three times (T2-4), followed by a second native saliva collection to assess sex hormone concentrations (for more detail, see Fricke et al. 2023). Participants then underwent another assessment of current mood and the second BART with a new balloon set. This protocol was in line with previous studies for both the dosages and the 45-min waiting period before task onset (Schwabe et al. 2010; 2012; Schwabe and Wolf 2013). Lastly, participants performed a second task not relevant to the current research question (approach-avoidance-conflict task; see Fricke et al. 2023), followed by a final saliva sample, vital signs, and mood assessments (T5) before being debriefed. The experiment lasted approximately 2 h and 40 min.

### Balloon analogue risk task and outcome parameters

The BART, originally developed by Lejuez et al. (2002), is a risk-taking task in which participants can pump up a virtual balloon by button click with the balloon increasing in size as visual feedback (see Fig. 1B). For each click, the monetary value of the balloon is increased by one cent. Participants need to decide how much they are willing to pump up the balloon before cashing out, as each click increases the risk of the balloon bursting (accompanied by visual and auditory cues of a bursting balloon). In this version of the task, which has been previously utilized by Kluen et al. (2017), 60 balloons are presented in three colors with three pseudo-randomly assigned degrees of associated risk. Differences in risk levels were realized by randomized sampling out of the numbers 1:16, 1:32, or 1:64 to determine at which click each of the 60 balloons could burst, resulting in a high, moderate, and low risk condition, respectively. Participants were not informed that balloon color was related to risk level. Since the BART was performed twice, two sets of colors were presented in counterbalanced order (set 1: blue, green, yellow; set 2: light blue, purple, orange). After completion of the second BART, participants were asked to indicate the color associated with each risk level for both BART assessments. Participants were incentivized to not burst balloons as they were told that the money they secured by cashing out would be added to their monetary compensation at the end of the study.

Our primary outcome measure of the BART were the average adjusted clicks, i.e., the average number of clicks excluding trials in which the balloon burst. This is a commonly used performance measure of the BART (Lejuez et al. 2002). In addition, we also investigated the percentage of burst balloons. In line with Kluen et al. (2017), we excluded trials in which the balloon burst on the first trial as participants were unable to affect this specific outcome. Outcome measures were recorded for each BART both globally and per risk level.

### Statistical analysis

Analyses regarding baseline group differences and the effectiveness of our pharmacological intervention have been carried out in Fricke et al. (2023) and will be reported in brief. Whether participants were able to determine the correct risk level per balloon color was assessed descriptively to probe whether explicit task knowledge may have influenced task behavior.

To analyze the effects of the pharmacological interventions and their interactions with gender on risk-taking, we conducted ANOVAs with the within-subject factors risk level and time (prior vs. post pharmacological intervention) and the between-subjects factors hydrocortisone, yohimbine, and gender for the outcome parameters average adjusted clicks and fraction of burst balloons. Findings were qualified by additional Bayesian ANOVAs to estimate the likelihood of the null or alternative hypothesis being true. For this, null models including only the within-subject variable risk level were defined and the Bayes factors (BFs) of all other respective models containing additional factors (i.e., time, hydrocortisone, yohimbine, and gender) and factor interactions compared against these null models. Bayes factors are reported in terms of the null model vs. the respective model if evidence favored the null model (1/BF), and were interpreted as suggested by Kass and Raftery (1995).

In addition, we correlated the assessed personality traits and baseline testosterone and estradiol concentrations with the outcome measures of the first BART to estimate the potential influences of aggression, anxiety, and sensation seeking, as well as sex hormones on behavioral risk-taking. We focused on the first BART for these correlational analyses to ensure that behavior and hormone concentrations were not affected by the pharmacological intervention or learning.

For analyses on the average adjusted clicks, two women (one in the hydrocortisone and one in the hydrocortisone + yohimbine group) had to be excluded as they had burst all high-risk balloons in both BART sessions. Additionally, five men (two in the control, two in the hydrocortisone, and one in the hydrocortisone + yohimbine group) had to be excluded from analyses involving the second BART as they had burst all high-risk balloons in that session.

For significant findings in the ANOVAs detailed above, the appropriate follow-up tests, including ANOVAs and Welch t-tests, were conducted. When sphericity was violated, we employed Greenhouse–Geisser correction. Post-hoc Bonferroni-Holm corrections for multiple testing were based on the number of separate post-hoc ANOVAs or t-tests per analysis. All reported p-values are two-tailed. All analyses were carried out in R (Version 4.4.0; R Core Team 2024), see osf.io/wrkh3.

## Results

The four experimental groups did not differ in age, body-mass index, or the investigated personality traits (anxiety, aggression, sensation seeking; see Online Resource Table S1). Regarding our pharmacological manipulation, hydrocortisone administration increased salivary cortisol concentrations as expected, leading to elevated cortisol concentrations in the hydrocortisone groups during the second, but not the first BART. Likewise, yohimbine induced SNS activation as evidenced by elevations in salivary alpha amylase activity during the second, but not the first BART and significant differences in blood pressure and heart rate over the five points of measurement (see Online Resource Figure S1). Subjective mood was unaffected by medication intake and participants were unable to differentiate active medication from placebo, indicating successful blinding (for details, see Fricke et al. 2023).

Participants struggled to identify the correct balloon colors for each risk level. Only 20% indicated all balloon colors correctly for the first BART, whereas 33% were able to correctly identify them for the second BART. The better performance in the second BART may be due to learning and recency as participants were asked to identify colors for both sessions after execution of the second BART. Regarding individual risk levels, participants were able to identify high-risk balloons best with accuracy rates of 42% (vs. 33% for low risk and 34% for moderate risk) in the first BART and 52% (vs. 45% and 42%, respectively) in the second BART. The overall low accuracy suggests limited reliance on explicit strategies based on balloon color.

### Effects of hydrocortisone, yohimbine, their interaction, and gender on risk-taking behavior

**Fig. 2:**
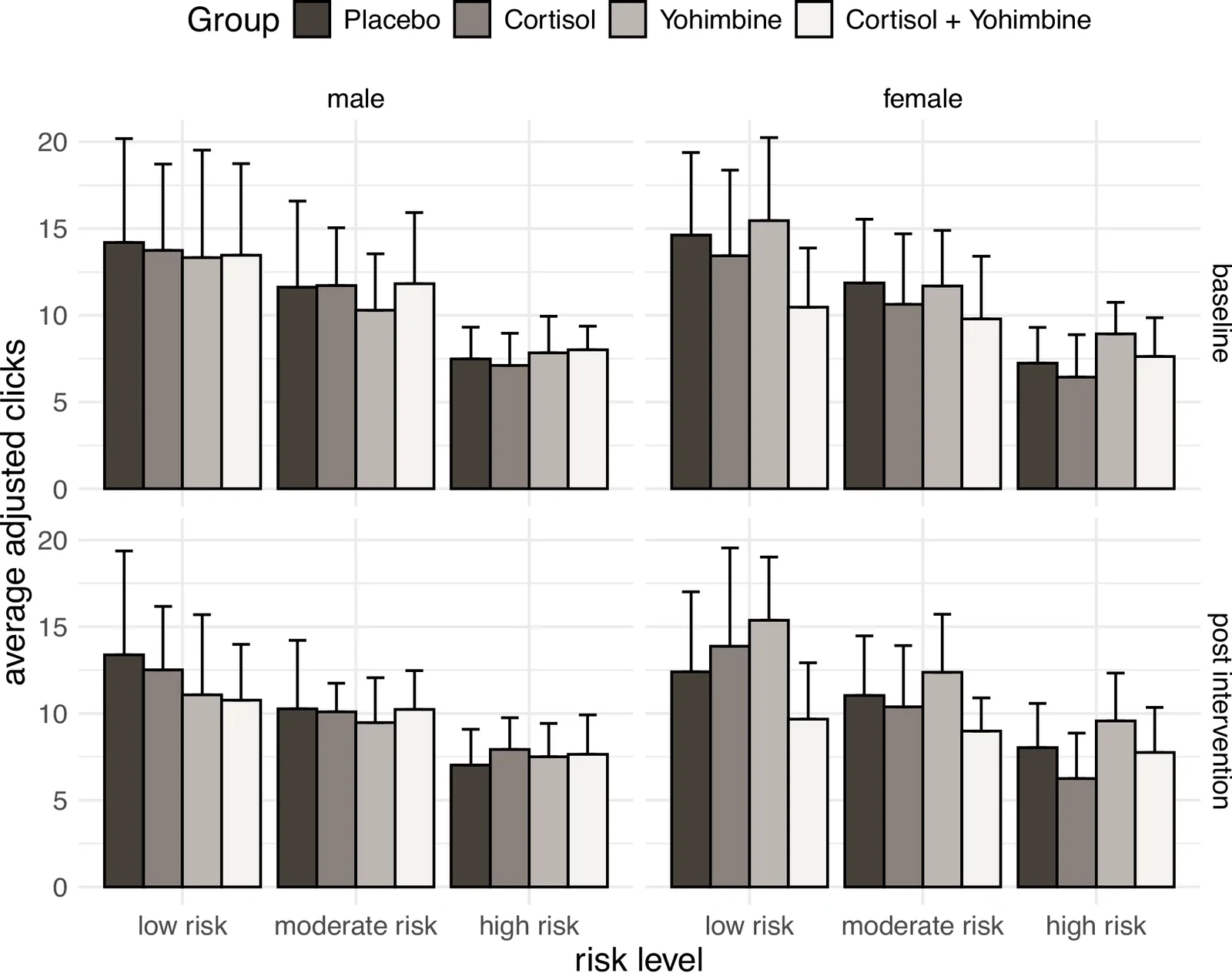
The average adjusted clicks (excluding trials, in which the balloon burst) in the BART balloon analogue risk task (mean ± SD) for men and women (separated by intervention group) prior to (top) and post pharmacological intervention (bottom)

**Fig. 3:**
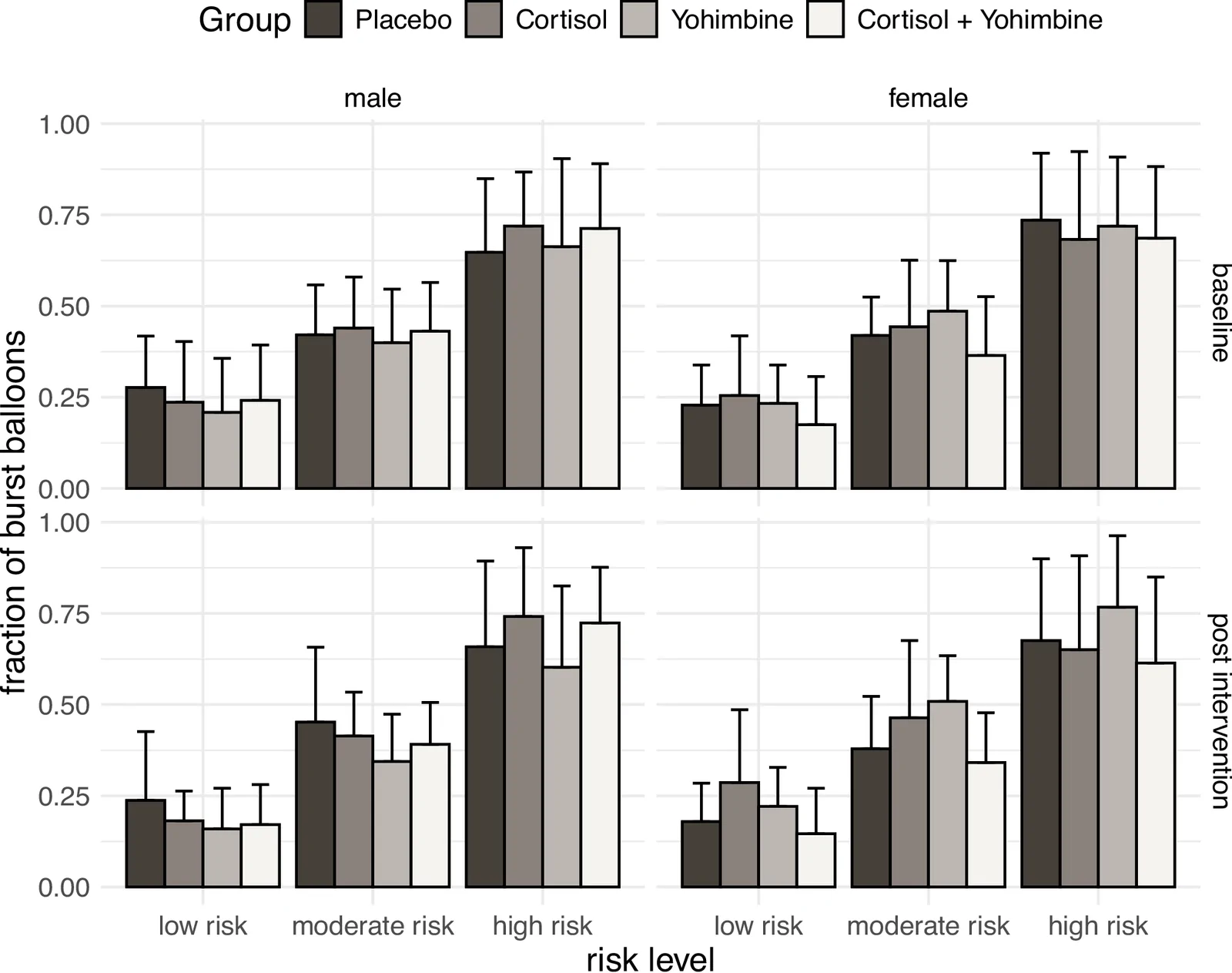
The fraction of balloons that burst in the balloon analogue risk task (mean ± SD) for men and women (separated by intervention group) prior to (top) and post pharmacological intervention (bottom)

As expected, participants showed strong differentiation across risk levels, with large effects for both BART outcome measures (average adjusted clicks: F_1.25,96.36_ = 121.072, *p* < 0.001, generalized η^2^ = 0.283, see Fig. 2; burst fraction: F_1.29,108.34_ = 731.546, *p* < 0.001, generalized η^2^ = 0.598, see Fig. 3). Participants were slightly more cautious in the second BART session, indicated by small but significant main effects of time (12.47 vs. 11.69; F_1,77_ = 4.872, *p* = 0.030, generalized η^2^ = 0.006; burst fraction: 0.44 vs. 0.43; F_1,84_ = 5.159, *p* = 0.026, generalized η^2^ = 0.003).

Our primary interest, however, was in interactive effects of pharmacological interventions and either time or time and gender to determine how hydrocortisone and yohimbine affected risk-taking behavior in the BART (for all other effects, see Online Resource Table S2).

For the average adjusted clicks, no interactions of time and the between-variables (hydrocortisone, yohimbine, and gender) were found (see Fig. 2). In addition, Bayes factors comparing the risk level only model to models including interactions of time with between-subjects factors indicated no substantial evidence for any of these alternative models, although some models were inconclusive in terms of whether they favored the null hypothesis (all models including time-by-medication, time-by-gender, and/or time-by-medication-by-gender interactions: BF ≤ 1.737; see Online Resource Table S3; for full list of model comparisons, see analyses at osf.io/wrkh3).

For the fraction of burst balloons, a four-way interaction involving yohimbine, hydrocortisone, gender, and time reached significance (F_1,84_ = 10.714, *p* = 0.002, generalized η^2^ = 0.006; see Fig. 3). Post-hoc analyses based on time did not qualify this interaction further, but may suggest an interaction of yohimbine, hydrocortisone, and gender in the second BART (F_1,85_ = 4.249, *p* = 0.084, generalized η^2^ = 0.035). However, all Bayesian models including interactions of time with either pharmacological intervention, gender, or both strongly or decisively favored the null hypothesis (1/BF ≥ 15.789), suggesting no additional explanatory power of medication over the effect of risk level in the BART (for full list of model comparisons, see data included in analyses at osf.io/wrkh3).

### Correlations of risk-taking behavior with personality traits and sex hormones

**Fig. 4:**
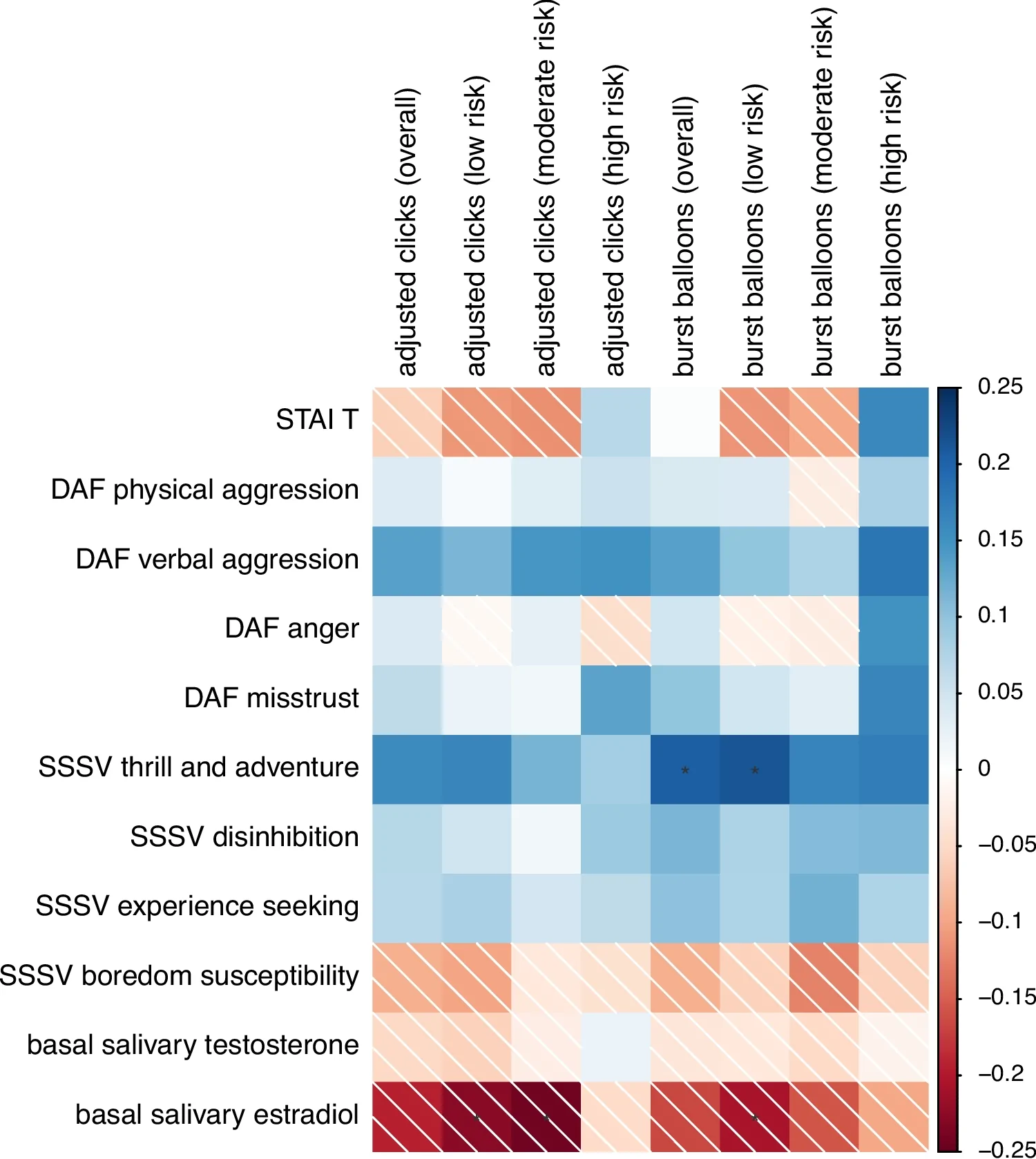
Correlation matrix of questionnaire scores and basal salivary sex hormones with balloon analogue risk task outcome measures (average adjusted clicks; fraction of burst balloons). Color intensity indicates strength of correlation, color and shade indicate direction of effect (blue/no shade = positively correlated; red/shaded = negatively correlated). p-values are uncorrected for multiple comparisons. * *p* <.05. STAI-T: State-Trait Anxiety Inventory – Trait; DAF: Deutscher Aggressionsfragebogen (German Aggression Questionnaire); SSSV: Sensation Seeking Scale

Of the tested variables, only the thrill and adventure subscale of the SSS-V and basal salivary estradiol correlated with risk-taking measures during the first BART (see Fig. 4 for an overview as well as Online Resource Figure S2 for scatterplots of significant correlations). The thrill and adventure subscale was positively correlated with the overall fraction of burst balloons (r_91_ = 0.206, *p* = 0.048) and the fraction of burst low-risk balloons (r_91_ = 0.213, *p* = 0.041), suggesting riskier behavior in individuals scoring higher on this sensation seeking scale. In contrast, estradiol was negatively correlated with the average adjusted clicks in the low- (r_90_ = -0.225, *p* = 0.031) and moderate-risk (r_90_ = -0.241, *p* = 0.021) balloon categories, and the fraction of burst balloons in the low-risk category (r_90_ = -0.206, *p* = 0.048). Importantly, these correlations would not hold if corrected for multiple testing and should therefore be interpreted carefully.

## Discussion

Being in a stressful situation can significantly impact decision-making by prioritizing reward gain, thus resulting in overall riskier decisions. While small effects of stress on risky decision-making have been meta-analytically supported (Starcke and Brand 2016), the underlying mechanisms remain less understood and current findings are in part contradictory. Understanding these mechanisms, however, could elucidate the etiology of stress-related disorders that involve risky decision-making and aid in identifying intervention targets. In this study, the effects of hydrocortisone and yohimbine, their interactions with one another, and with gender were examined for their role in risky decision-making. Four groups of healthy participants received either hydrocortisone, yohimbine, both, or placebo in-between two runs of an established task probing for risky-decision making. Additionally, the influences of trait anxiety, aggression, and sensation seeking as well as endogenous testosterone and estradiol prior to the pharmacological intervention were analyzed, as these factors were identified to potentially affect risky decision-making, but remain under-investigated.

### No expected effects of hydrocortisone, yohimbine, or gender on risky decision-making

Hydrocortisone effectively increased salivary cortisol concentration, while yohimbine increased salivary alpha amylase activity and altered blood pressure and heart rate, indicating activation of the HPA axis and SNS, respectively. However, the hypothesis that hydrocortisone and yohimbine combined would increase risk-taking was not supported. Likewise, an earlier cross-sectional finding of riskier decision making in males after cortisol administration (Kluen et al. 2017) was also not replicated (note, however, that we corrected more strictly for multiple testing). Bayesian analyses yielded little to no evidence for effects of hydrocortisone, yohimbine, or their interaction with gender, (Kass and Raftery 1995), further supporting the absence of meaningful drug effects during the second BART. Overall, the data presented here did not show moderate or strong effects of medication or their interaction with gender on BART outcome measures.

Our findings are in line with studies suggesting that the effects of pharmacological stimulation of stress pathways differ from or are not as easily interpretable as the induction of psychosocial stress, which often – but not always – elicits small increases in risk-taking (Starcke and Brand 2016; of note: this meta analysis also found no effect of gender). A recent systematic review on pharmacological SNS and HPA axis manipulations by Sarmiento et al. (2024) suggests that activation of the SNS in isolation likely does not significantly impact risky decision-making. For HPA axis modulation, the authors of the meta-analysis suggested that time between pharmacological intervention and task may be relevant as effects of increased risky decision-making were only found in studies with drug-task intervals of at least 60 min (Sarmiento et al. 2024). This could explain our null findings as our participants performed the second BART 45 min after administration of the pharmacological intervention. Mechanistically, this could be fitting to genomic corticosteroid effects which are expected to develop more slowly than non-genomic corticosteroid effects (Hermans et al. 2014; Joels et al. 2013). Additionally, given the absorption time of hydrocortisone resulting in peak concentrations after approximately 1 h (Hindmarsh and Charmandari 2015), our null results could also indicate that cortisol effects on decision-making depend on its concentration. We suggest that the temporal distance between intervention and task onset should thus be carefully considered or systemically manipulated in future studies. Importantly, the longer temporal delay may be particularly important to studies with pharmacological manipulations as Starcke and Brand (2016) found a small, but significant meta-analytic effect of stress induction on decision-making despite only including studies with a stress-task interval < 60 min.

Regarding pharmacological manipulations of the HPA axis in particular, it should be noted that hydrocortisone is an unspecific agonist for both receptor types of cortisol and one study found altered risk taking after stimulating specifically the mineralocorticoid receptor (Deuter et al. 2017). Thus, next to timing and concentration, also the specificity of the pharmacological agent may be relevant to consider.

For the combined administration of hydrocortisone and yohimbine, heterogenous findings regarding risky decision-making have been described in previous studies. Dosages in our study were similar to the discussed studies (identical dosage: Kluen et al. 2017; Margittai et al. 2018; half the dosage, i.e., 10 mg each: Metz et al. 2020), making it unlikely that differences in findings were only dosage-related. However, differences in results may be due to gender composition (Margittai et al. 2018; and Metz et al. 2020 used male only samples) or to timing, given that Kluen et al. (2017) used a longer drug-task interval of 85 min. Especially in the context of administrating both, hydrocortisone and yohimbine, to investigate interaction effects, timing of administration may be particularly intricate given their different pharmacokinetic profiles (Hindmarsh and Charmandari 2015; Owen et al. 1987).

Another relevant aspect is that subjective stress levels remained unaffected by our pharmacological manipulation. This is a frequent finding as pharmacological manipulations of the stress systems are often not subjectively considered as stress by participants (Sarmiento et al. 2024), which may therefore have diminished pharmacological effects on task performance. An explanation could be that the negative affect, which accompanies stress-induced activations of the SNS and the HPA axis in real-life, emphasizes increased risk-taking behaviors (e.g., von Helversen and Rieskamp 2020). Interestingly, the meta-analysis of Starcke and Brand (2016) also indicates that certain stress induction procedures (e.g., the cold pressor test), which result in robust physiological stress system activation but not necessarily subjective stress, induce a much smaller effect on decision-making compared to psychosocial stressors which typically induce both physiological and subjective stress. A counterexample, however, is the study by Kluen et al. (2017) in which effects of hydrocortisone in the BART were found despite not influencing subjective mood, suggesting that mood effects are not always a necessity for effects of stress mediators on risk-taking.

Finally, from a task-design perspective, it may be worthwhile to explore different operationalizations of risky decision-making. For instance, the BART was found to be rather test–retest reliable (e.g., Buelow and Barnhart 2018; White et al. 2008), indicating that it measures a trait construct, which may be less susceptible to pharmacological interventions. Yet again, several other studies did find effects of experimental manipulations on behavior in the BART.

### Prior task experience may obscure effects of stress mediators and gender

One difference between several prior studies investigating the effect of stress mediators on risky behavior in the BART and ours is the inclusion of a baseline BART before the pharmacological intervention. The major advantage of including a baseline BART assessment is the increase in statistical power to detect medication effects by partitioning out variance resulting from baseline interindividual differences. However, it is possible that including a baseline BART may have led to adaptions to the task in the second task run. Time was indeed a significant predictor of both investigated outcome measures (albeit with small effect size), implying that participants performed slightly more cautious the second time around. However, as pharmacological intervention did not interact significantly with time or was not qualified post-hoc, differences between the first and second BART appear to be independent of the stress mediators manipulated here. One study suggests that stress effects on BART performance may be masked by task familiarity. More specifically, Nowacki et al. (2019) exposed healthy female participants to the BART on two separate testing days, once 80 min after a psychosocial stress induction and once after a non-stressful control procedure. Stress effects were only found when the BART was novel, i.e., on the first testing day, leading to decreased risk-taking. Furthermore, another study investigated the BART by having healthy male participants (of note: only 9 participants were examined) perform a baseline BART, and two further BARTs (once after 50 mg cortisol intake, once after a placebo), each at least two days apart. The authors found significantly increased risk-taking in the cortisol session compared to baseline, but not compared to the placebo session (Robertson et al. 2016), suggesting that the influences of a baseline BART may be dependent on the time interval between BARTs or the higher dosage in comparison to our study.

### Sensation seeking and estradiol concentrations may influence risky decision-making

Contrary to our expectation, we found only limited evidence that the selected personality traits, namely trait anxiety, aggression, and sensation seeking, as well as baseline sex hormone concentrations, influence risky decision-making as assessed with the BART. Most correlations we found involved outcome measures in the low-risk condition, in which detecting differences may have been easier as the decision space at a 1/64 chance of bursting the balloon was twice and four times as large as for moderate- and high-risk trials, respectively. More specifically, we found the thrill and adventure subscale of the SSS-V to be positively linked to an increased number of burst balloons, therefore indicating higher risk-taking. The finding is in line with the literature as a meta-analysis suggests small to moderate effects of sensation seeking on risk-taking in the BART (Lauriola et al. 2014). The previously reported small effect of decreases in risk-taking in the BART correlating with higher trait anxiety (Huo et al. 2020) was not found. Neither were links between aggression and increased risk-taking which have been suggested in studies using other paradigms probing risk-taking (Kuin et al. 2015). Then again, it should be noted that larger sample sizes or a more heterogeneous sample may have been needed to detect presumably small effects of interindividual trait differences in our study.

Regarding sex hormone concentrations, we found higher levels of salivary estradiol to be associated with reduced risk-taking, while salivary testosterone did not correlate with risk-taking. These findings are surprising as meta-analytical evidence suggests small positive correlations for both estradiol and testosterone, albeit less clear for estradiol when sample composition (male only, female only, or mixed) was taken into account (Kurath and Mata 2018). Overall, as our correlations were not corrected for multiple testing, it is possible that the found correlations are spurious. In addition, previously reported effects were small in size, possibly highlighting the need for larger or different samples (e.g., extreme group comparisons) to achieve adequate power. For our findings, it is especially important to keep in mind that our participant sample was likely to be rather homogeneous as participants were selected for a specific age range as well as physical and psychological health. This may have reduced interindividual differences, making it possible that effects are more easily found in the general population or more heterogenous samples.

## Conclusion

In conclusion, the administration of hydrocortisone, yohimbine, or both combined led to the expected changes in HPA axis and SNS activity, but not to the hypothesized effects on risk-taking. Especially the combination of both medications failed to replicate stress effects on risk-taking (Starcke and Brand 2016), although including the baseline session should have provided us with sufficient statistical power. Likewise, we did not replicate stronger risk taking in men after hydrocortisone administration (Kluen et al. 2017). This suggests that the effects of these stress mediators on human risk-taking, in pharmacological application or as part of the natural stress response, are complex. They may be affected not only by interindividual differences between participants, e.g., gender, but also experimental variables such as timing of medication (in general and perhaps differently based on medication, when combined), dosage of medication, receptor affinity, and task familiarity.

## Supplementary Information

Below is the link to the electronic supplementary material.

Supplementary file1 (PDF 405 KB)

## Data Availability

Data and analyses are available at osf.io/wrkh3.
