# Supplementary material for: Risky decision-making in the balloon analogue risk task – the role of noradrenaline and cortisol
Source: Psychopharmacology (Berl). 2025 Nov 10;243(8):1943–54. doi: 10.1007/s00213-025-06943-3 (PMC13506600; doi:10.1007/s00213-025-06943-3)
Supplement: Supplementary file 1 [file 213_2025_6943_MOESM1_ESM.pdf]

Supplemental Information

Table S1. Sample Characteristics

	PP (N=24)	CP (N=23)	YP (N=23)	CY (N=24)	F-value	p value
**age**					0.764	.517[1]
Mean (SD)	25.42 (5.12)	24.78 (4.24)	23.57 (3.62)	24.25 (4.30)		
Lower CI (95%)	23.25	22.95	22.00	22.44		
Upper CI (95%)	27.58	26.62	25.13	26.06		
**body-mass-index**					0.945	.423[1]
Mean (SD)	23.05 (2.12)	22.59 (2.48)	22.38 (1.81)	22.07 (1.82)		
Lower CI (95%)	22.16	21.52	21.59	21.31		
Upper CI (95%)	23.95	23.66	23.16	22.84		
**trait anxiety**					0.651	.584[1]
Mean (SD)	34.54 (7.19)	38.04 (9.10)	36.61 (10.22)	35.88 (8.40)		
Lower CI (95%)	31.51	34.11	32.19	32.33		
Upper CI (95%)	37.58	41.98	41.03	39.42		
**physical aggression**					0.468	.705[1]
Mean (SD)	13.17 (3.60)	14.22 (3.91)	13.09 (3.13)	13.29 (4.04)		
Lower CI (95%)	11.65	12.53	11.73	11.59		
Upper CI (95%)	14.68	15.91	14.44	15.00		
**verbal aggression**					0.969	.411[1]
Mean (SD)	10.04 (2.46)	10.96 (2.6)	11.35 (3.02)	10.79 (2.7)		
Lower CI (95%)	9.00	9.83	10.04	9.65		
Upper CI (95%)	11.08	12.08	12.66	11.93		
**anger**					0.104	.957[1]
Mean (SD)	12.29 (3.61)	12.83 (3.83)	12.43 (3.42)	12.71 (3.85)		
Lower CI (95%)	10.77	11.17	10.95	11.08		
Upper CI (95%)	13.81	14.48	13.91	14.33		
**mistrust**					0.617	.606[1]
Mean (SD)	13.08 (4.09)	14.30 (3.69)	14.26 (3.51)	13.54 (3.27)		
Lower CI (95%)	11.36	12.71	12.74	12.16		
Upper CI (95%)	14.81	15.90	15.78	14.92		
**sensation seeking**					1.496	.221[1]
Mean (SD)	20.00 (6.34)	22.35 (5.22)	21.70 (4.91)	23.04 (4.11)		
Lower CI (95%)	17.32	20.09	19.57	21.31		
Upper CI (95%)	22.68	24.60	23.82	24.78		

Note. Degrees of freedom are 3 and 90 for all tests. Groups: placebo (PP), hydrocortisone (CP), yohimbine (YP), hydrocortisone and yohimbine (CY). [1]Linear Model ANOVA. Reused with permission from Fricke, K., Alexander, N., Jacobsen, T. et al. The effects of hydrocortisone and yohimbine on human behavior in approach-avoidance conflicts. Psychopharmacology 240, 1705–1717 (2023). https://doi.org/10.1007/s00213-023-06396-6. and covered under a Creative Commons Attribution 4.0 International License

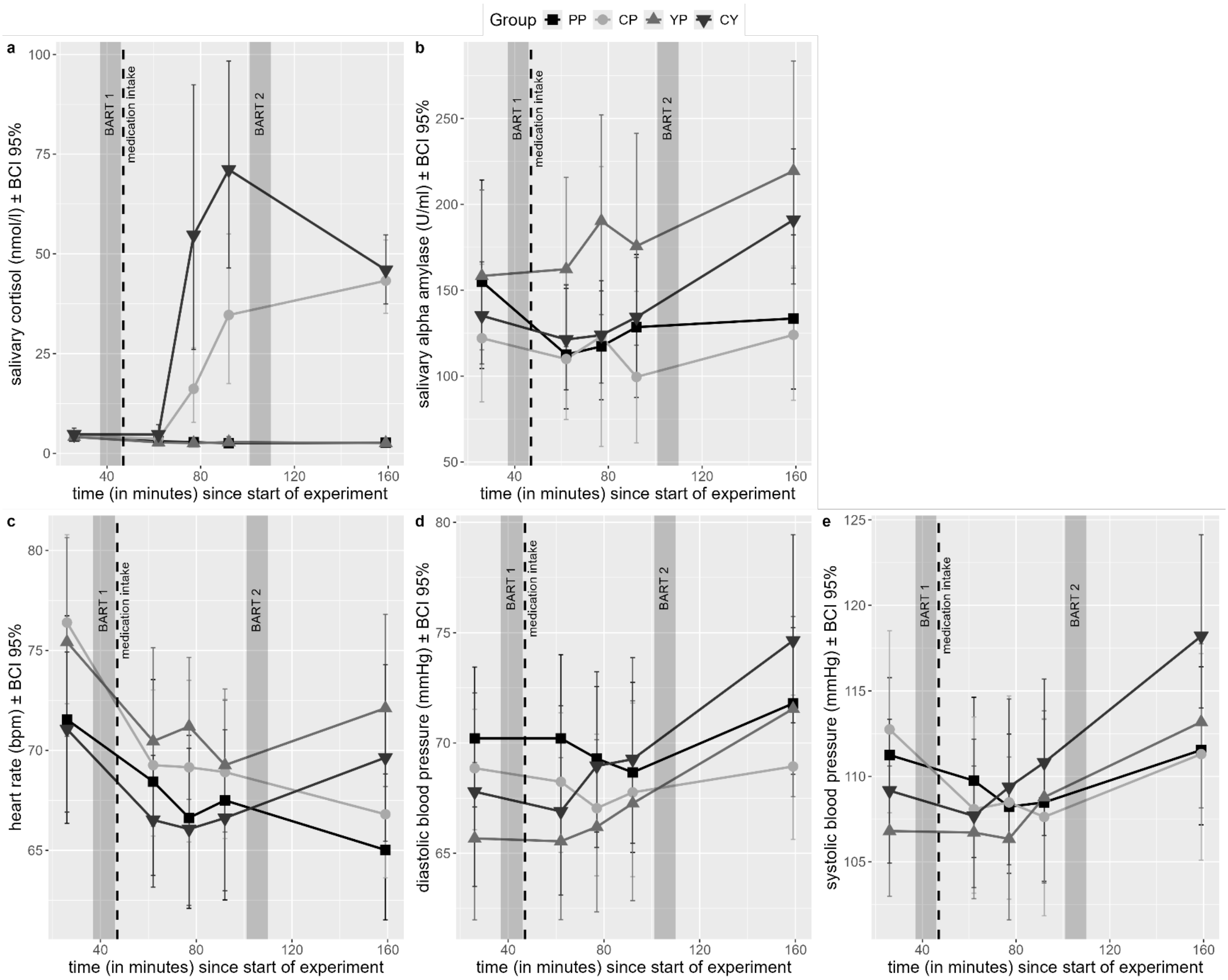

Figure S2. Measures of (a) salivary cortisol concentration, (b) salivary alpha amylase activity, (c) heart rate, (d) diastolic blood pressure, and (e) systolic blood pressure over time. Dotted line indicates time of medication intake. Grey overlay indicates duration of the first and second Balloon Analogue Risk Tast (BART). Error bars represent bootstrapped 95% confidence intervals (BCI). Groups: placebo (PP), hydrocortisone (CP), yohimbine (YP), hydrocortisone and yohimbine (CY). Adapted with permission from Fricke, K., Alexander, N., Jacobsen, T. et al. The effects of hydrocortisone and yohimbine on human behavior in approach-avoidance conflicts. Psychopharmacology 240, 1705–1717 (2023). https://doi.org/10.1007/s00213-023-06396-6. and covered under a Creative Commons Attribution 4.0 International License. For statistical details, please refer to the original article.

Table S2. Full ANOVA result tables for dependent variables average adjusted clicks and percentage burst balloons.

**dependent variable: average adjusted clicks**	
Factors	Statistic
gender	$F(1, 77) = 0.060$, $p = 0.807$, generalized $\eta^2 = 0.000$
yohimbine	$F(1, 77) = 0.111$, $p = 0.740$, generalized $\eta^2 = 0.001$
cortisol	$F(1, 77) = 2.825$, $p = 0.097$, generalized $\eta^2 = 0.020$
**time**	**$F(1, 77) = 4.872$, $p = 0.030$, generalized $\eta^2 = 0.006$**
**risk type**	**$F(1.25, 96.36) = 121.072$, $p < 0.001$, generalized $\eta^2 = 0.283$**
gender*yohimbine	$F(1, 77) = 0.407$, $p = 0.525$, generalized $\eta^2 = 0.003$
gender*cortisol	$F(1, 77) = 2.974$, $p = 0.089$, generalized $\eta^2 = 0.021$
yohimbine*cortisol	$F(1, 77) = 0.471$, $p = 0.494$, generalized $\eta^2 = 0.003$
gender*time	$F(1, 77) = 2.631$, $p = 0.109$, generalized $\eta^2 = 0.003$
yohimbine*time	$F(1, 77) = 0.302$, $p = 0.585$, generalized $\eta^2 = 0.000$
cortisol*time	$F(1, 77) = 0.074$, $p = 0.786$, generalized $\eta^2 = 0.000$
gender*risk type	$F(1.25, 96.36) = 0.056$, $p = 0.864$, generalized $\eta^2 = 0.000$
**yohimbine*risk type**	**$F(1.25, 96.36) = 3.855$, $p = 0.043$, generalized $\eta^2 = 0.012$**
cortisol*risk type	$F(1.25, 96.36) = 1.162$, $p = 0.296$, generalized $\eta^2 = 0.004$
**time*risk type**	**$F(1.86, 143) = 4.992$, $p = 0.010$, generalized $\eta^2 = 0.006$**
gender*yohimbine*cortisol	$F(1, 77) = 1.781$, $p = 0.186$, generalized $\eta^2 = 0.013$
gender*yohimbine*time	$F(1, 77) = 0.991$, $p = 0.323$, generalized $\eta^2 = 0.001$
gender*cortisol*time	$F(1, 77) = 0.128$, $p = 0.722$, generalized $\eta^2 = 0.000$
yohimbine*cortisol*time	$F(1, 77) = 1.118$, $p = 0.294$, generalized $\eta^2 = 0.001$
gender*yohimbine*risk type	$F(1.25, 96.36) = 0.133$, $p = 0.773$, generalized $\eta^2 = 0.000$
gender*cortisol*risk type	$F(1.25, 96.36) = 0.285$, $p = 0.647$, generalized $\eta^2 = 0.001$
yohimbine*cortisol*risk type	$F(1.25, 96.36) = 1.705$, $p = 0.195$, generalized $\eta^2 = 0.006$
gender*time*risk type	$F(1.86, 143) = 0.782$, $p = 0.451$, generalized $\eta^2 = 0.001$
yohimbine*time*risk type	$F(1.86, 143) = 0.521$, $p = 0.582$, generalized $\eta^2 = 0.001$
cortisol*time*risk type	$F(1.86, 143) = 0.532$, $p = 0.576$, generalized $\eta^2 = 0.001$
gender*yohimbine*cortisol*time	$F(1, 77) = 0.757$, $p = 0.387$, generalized $\eta^2 = 0.001$
gender*yohimbine*cortisol*risk type	$F(1.25, 96.36) = 1.979$, $p = 0.159$, generalized $\eta^2 = 0.006$
gender*yohimbine*time*risk type	$F(1.86, 143) = 0.508$, $p = 0.589$, generalized $\eta^2 = 0.001$
gender*cortisol*time*risk type	$F(1.86, 143) = 1.860$, $p = 0.162$, generalized $\eta^2 = 0.002$
yohimbine*cortisol*time*risk type	$F(1.86, 143) = 0.655$, $p = 0.510$, generalized $\eta^2 = 0.001$
gender*yohimbine*cortisol*time*risk type	$F(1.86, 143) = 1.720$, $p = 0.185$, generalized $\eta^2 = 0.002$
**dependent variable: percentage of burst balloons**	
Factors	Statistic
gender	$F(1, 84) = 0.012$, $p = 0.914$, generalized $\eta^2 = 0.000$
yohimbine	$F(1, 84) = 0.655$, $p = 0.421$, generalized $\eta^2 = 0.006$
cortisol	$F(1, 84) = 0.095$, $p = 0.759$, generalized $\eta^2 = 0.001$
**time**	**$F(1, 84) = 5.159$, $p = 0.026$, generalized $\eta^2 = 0.003$**
**risk type**	**$F(1.29, 108.34) = 731.546$, $p < 0.001$, generalized $\eta^2 = 0.598$**
gender*yohimbine	$F(1, 84) = 0.249$, $p = 0.619$, generalized $\eta^2 = 0.002$
gender*cortisol	$F(1, 84) = 1.064$, $p = 0.305$, generalized $\eta^2 = 0.009$
yohimbine*cortisol	$F(1, 84) = 0.340$, $p = 0.561$, generalized $\eta^2 = 0.003$
gender*time	$F(1, 84) = 0.175$, $p = 0.677$, generalized $\eta^2 = 0.000$

yohimbine*time	F(1, 84) = 1.941, p = 0.167, generalized η^2 = 0.001
cortisol*time	F(1, 84) = 0.115, p = 0.736, generalized η^2 = 0.000
gender*risk type	F(1.29, 108.34) = 0.113, p = 0.801, generalized η^2 = 0.000
yohimbine*risk type	F(1.29, 108.34) = 1.058, p = 0.324, generalized η^2 = 0.002
cortisol*risk type	F(1.29, 108.34) = 0.304, p = 0.640, generalized η^2 = 0.001
time*risk type	F(2, 168) = 1.495, p = 0.227, generalized η^2 = 0.001
gender*yohimbine*cortisol	F(1, 84) = 2.355, p = 0.129, generalized η^2 = 0.020
gender*yohimbine*time	F(1, 84) = 3.238, p = 0.076, generalized η^2 = 0.002
gender*cortisol*time	F(1, 84) = 0.245, p = 0.622, generalized η^2 = 0.000
yohimbine*cortisol*time	F(1, 84) = 0.908, p = 0.343, generalized η^2 = 0.001
gender*yohimbine*risk type	F(1.29, 108.34) = 0.611, p = 0.476, generalized η^2 = 0.001
**gender*cortisol*risk type**	**F(1.29, 108.34) = 4.974, p = 0.020, generalized η^2 = 0.010**
yohimbine*cortisol*risk type	F(1.29, 108.34) = 0.727, p = 0.429, generalized η^2 = 0.001
gender*time*risk type	F(2, 168) = 2.342, p = 0.099, generalized η^2 = 0.002
yohimbine*time*risk type	F(2, 168) = 0.082, p = 0.921, generalized η^2 = 0.000
cortisol*time*risk type	F(2, 168) = 0.131, p = 0.878, generalized η^2 = 0.000
**gender*yohimbine*cortisol*time**	**F(1, 84) = 10.714, p = 0.002, generalized η^2 = 0.006**
gender*yohimbine*cortisol*risk type	F(1.29, 108.34) = 1.749, p = 0.188, generalized η^2 = 0.004
gender*yohimbine*time*risk type	F(2, 168) = 0.871, p = 0.420, generalized η^2 = 0.001
gender*cortisol*time*risk type	F(2, 168) = 1.926, p = 0.149, generalized η^2 = 0.002
yohimbine*cortisol*time*risk type	F(2, 168) = 0.173, p = 0.842, generalized η^2 = 0.000
gender*yohimbine*cortisol*time*risk type	F(2, 168) = 0.896, p = 0.410, generalized η^2 = 0.001

Frequentist ANOVA statistical results for both outcome measures of the Balloon Analogue Risk Task. Bold text indicates significance.

Table S3. Bayes factors for factor combinations of uncertain evidence for the null model against the model including interactions of time with hydrocortisone, yohimbine, and/or gender for the average adjusted clicks

Factors	1/BF	Error (in %)
time + risk type + yohimbine + time:yohimbine + risk type:yohimbine + VPN	0.576	0.014
time + risk type + time:risk type + yohimbine + time:yohimbine + risk type:yohimbine + VPN	1.004	0.021
time + risk type + cortisol + yohimbine + time:yohimbine + risk type:yohimbine + VPN	1.901	0.016
time + risk type + yohimbine + time:yohimbine + risk type:yohimbine + gender + VPN	2.303	0.014
time + risk type + time:risk type + yohimbine + risk type:yohimbine + gender + time:gender + VPN	2.469	0.016
time + risk type + cortisol + time:cortisol + yohimbine + risk type:yohimbine + VPN	2.476	0.016
time + risk type + yohimbine + risk type:yohimbine + gender + time:gender + yohimbine:gender + VPN	3.146	0.017

Displayed are only factor combinations not exceeding the threshold of substantial evidence (BF > 3.2) in favor of the null hypothesis (risk level + participant) for adjusted average clicks including the interaction of time with gender, hydrocortisone or yohimbine. All other factor combinations resulted in substantial evidence in favor of the null hypothesis. 1/BF represents Bayes factors in terms of the null model vs. the model including the listed factors.

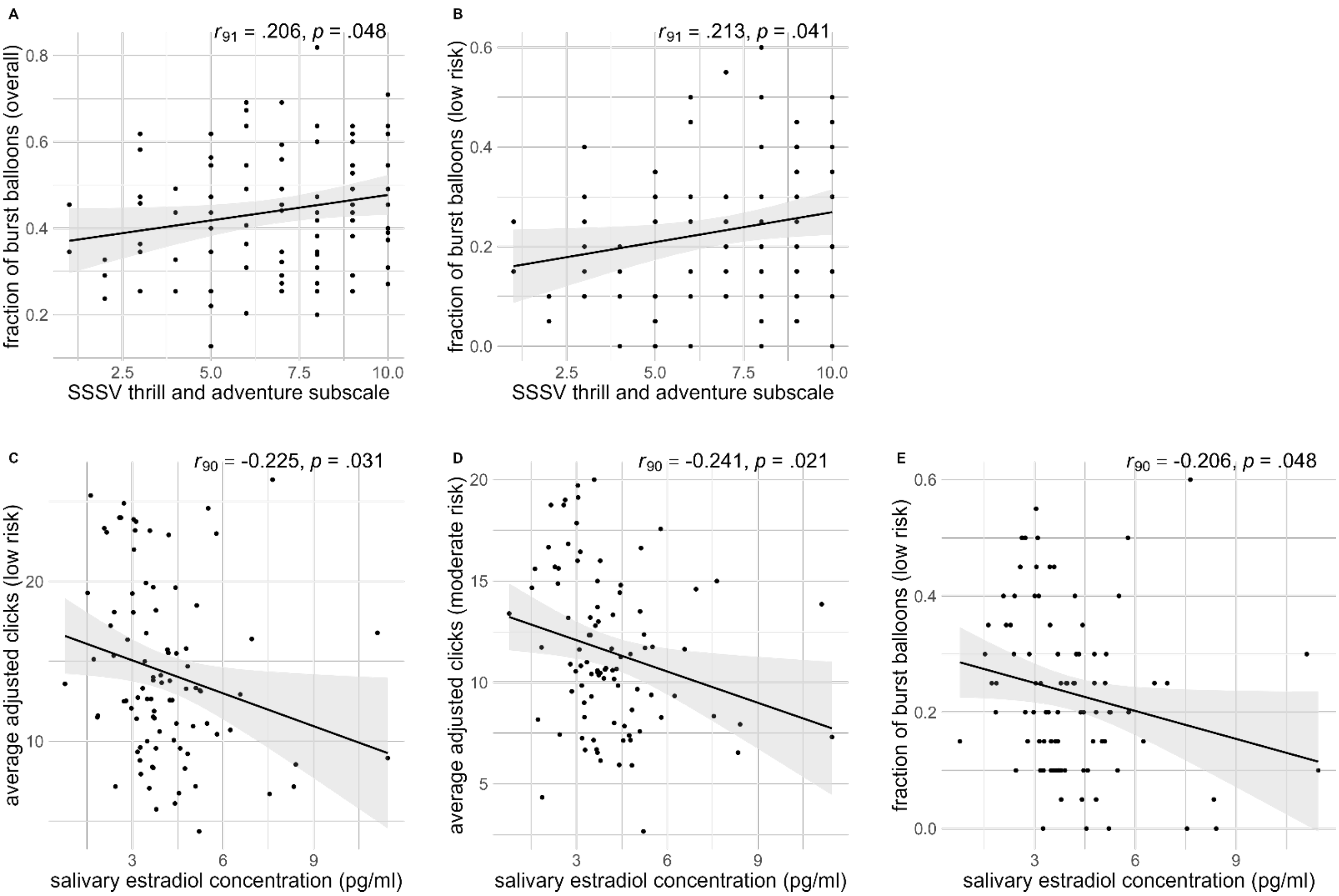

Figure S2. Scatterplots of nominally significant correlations of the SSS-V subscale thrill and adventure seeking with the fraction of A) overall and B) low-risk burst balloons as well as correlations of salivary estradiol concentrations with C) the average adjusted clicks in low-risk trials, D) the average adjusted clicks in moderate risk trials and E) the fraction of low-risk burst balloons. Pearson correlations are depicted above each subplot. Line indicates linear regression over all data points with 95% confidence interval.
